# Clinical characteristics of MOG antibody disease: two case reports

**DOI:** 10.1007/s10072-021-05122-4

**Published:** 2021-02-10

**Authors:** Yujia Guan, Zunwei Zhang, Mingming Li, Miao Shi, Hui Deng

**Affiliations:** Department of Neurology, First Hospital of Jilin University, Xinmin Street 1#, Changchun, 130021 China

## Introduction

Serum myelin oligodendrocyte glycoprotein (MOG) antibody-mediated idiopathic inflammatory demyelinating disease (IIDDs), a type of central nervous system demyelinating disease with MOG antibodies positive in serum, referred to as MOG antibody disease. This is different from multiple sclerosis (MS) or optic neuromyelitis lineage disease (NMOSD) because of its relatively unique clinical features, imaging findings, and special biomarkers, and it is now considered to be an independent disease. Disease syndrome mainly includes single or bilateral optic neuritis (ON), long-segment transverse myelitis (LETM), transverse myelitis (TM), optic neuritis plus transverse myelitis (ON+TM)/AQP4 antibody-negative NMOSD, and acute disseminated encephalomyelitis (ADEM) [1]. In order to further understand the clinical characteristics of MOG antibody disease, we present two patients with MOG antibody disease admitted in our hospital.

## Case report

**Fig. 1:**
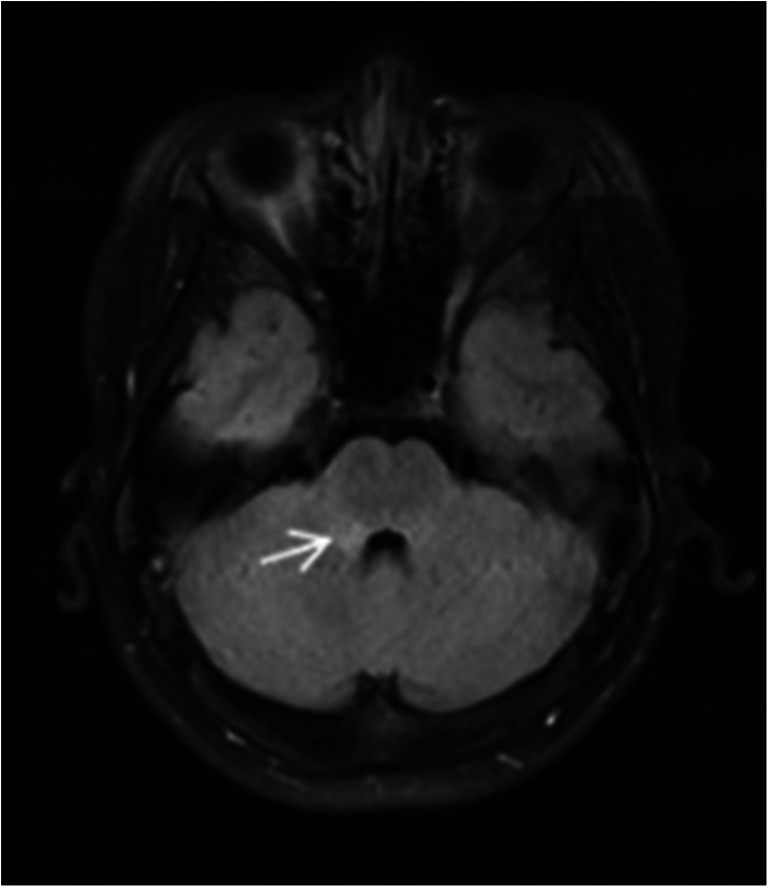
case 1: Brain MRI shouwed lesion in right brachium pontis on Flair on admission

**Fig. 2:**
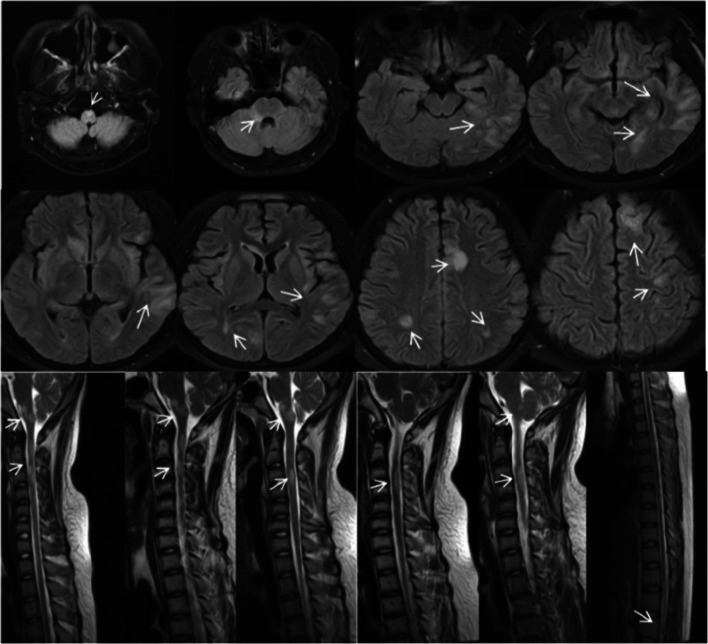
case 1: Brain MRI showed lesions in the bilateral frontal, apical, temporal, occipital cerebral gyrus, bilateral insular, right brachium pontis, medulla, and corpus callosum on Flair after progression of the disease. Spinal MRI showed lesions in the medulla oblongata, C2-T1, T4, T7, T11- T12, and spinal conus on T2 after progression of the disease

**Fig. 3:**
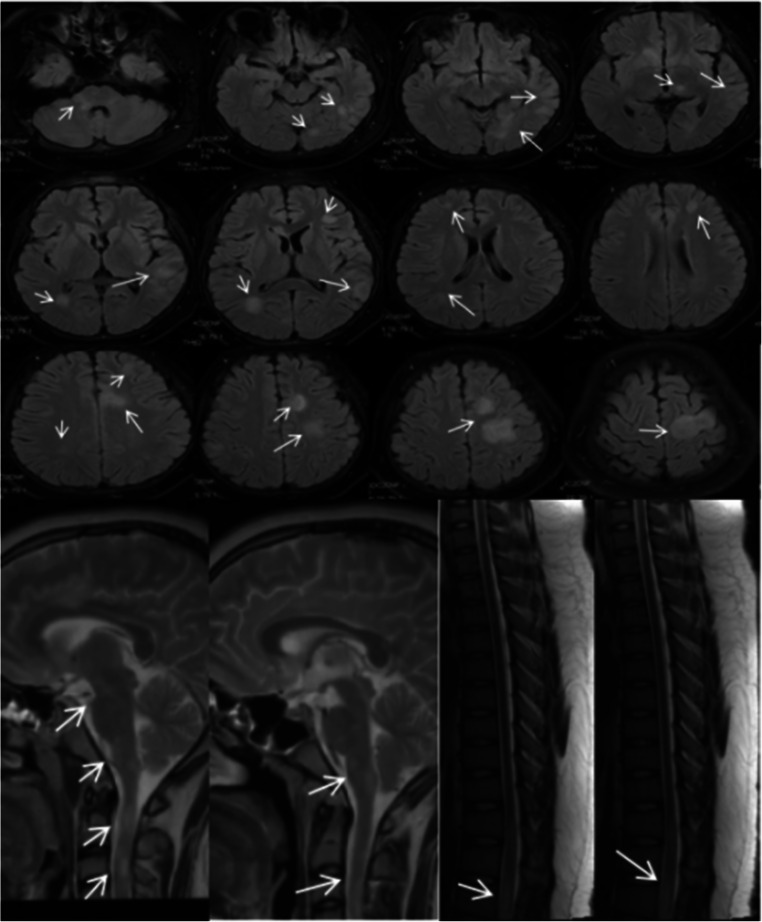
case 1: Brain MRI after symptom recurrence showed multiple intracranial lesions on Flair. Spinal MRI after symptom recurrence showed lesions in the medulla oblongata, spinal, and spinal conus on T2

A 15-year-old boy was admitted to our hospital because of a headache for 2 weeks, episodes of convulsions with fever for 3 days. His highest body temperature was 39.2 °C. At the time of admission, neurological examination revealed clear consciousness and speech, normal cranial nerve, grade 5 of limbs muscle strength on Muscle Strength Grading Scale, normal sense, unstable left finger-nose test and heel-knee-shin test, positive Babinski’s sign in left, suspiciously positive stiff-neck, and negative Kernig sign. After admission, the first cerebrospinal fluid (CSF) examination showed a pressure of 300 mm H2O, colorless and transparent cerebrospinal fluid, pleocytosis of 249×10^6^/L (multinuclear cell 0.1, mononuclear cell 0.9), normal concentration of protein, glucose, and chloride. The analysis of ANA, dsDNA Ab, ANCA, APL, erythrocyte sedimentation rate, C-reactive protein, thyroid function, tumor markers, HIV, syphilis, and hepatitis B virus were all normal. Brain MRI (1.5T) found the T1 hypointense, T2 and Flair hyperintense lesion in right brachium pontis (Fig. 1). MRV showed no obvious abnormality. 24-hour EEG showed non-specific slow wave in the frontal area. Within 15 days of admission, the condition continued to progress. The patient gradually appeared dizziness, emotional irritability, urinary incontinence, binocular vision loss only light sense, disorder of binocular movement, spontaneous rotatory nystagmus, dysarthria, dysphagia, grade 4 of the left upper limb on Muscle Strength Grading Scale, grade 3 of the right upper limb on Muscle Strength Grading Scale, grade 1 of lower limbs on Muscle Strength Grading Scale, low muscle tension, tendon jerk weakened, the sensory of pain reduction below L2, unstable left finger nose test, positive Babinski’s sign in left, neutral Babinski sign in right, no stiff-neck, and negative Kernig sign. Fundus examination showed binocular optic neuropathy. After the disease aggravated, we reviewed a cerebrospinal fluid examination and showed a pressure of 360 mm H2O, protein concentration of 0.53 g/L, pleocytosis of 164×10^6^/L (multinuclear 0.02, mononuclear 0.98), normal concentration of glucose and chloride, immunoglobulin G of 39.3 mg/L, and negative oligoclonal band. The AQP-4 antibody was negative in serum and cerebrospinal fluid. But the MOG antibody IgG was positive in serum (both RIA and CBA method positive). The review of Brain MRI (1.5T) showed that the lesions were significantly increased compared with that before 15 days. There were lesions in the bilateral frontal, apical, temporal, occipital cerebral gyrus, bilateral insular, right brachium pontis, medulla, and corpus callosum. A little gadolinium enhancement was found in some lesions (Fig. 2). Spinal MRI showed lesions in the medulla oblongata, C2-T1, T4, T7, T11-12, and spinal conus (Fig. 2). Spot-like gadolinium enhancement was found in lesions. After admission, the patient was given 2 g/kg of Human Immunoglobulin (PH4) (5 times) and steroids pulse. Methylprednisolone was given from 1000 mg/day, and halved every three days until 40 mg/day; then, the methylprednisolone was changed to oral, and reduced one tablet every 15 days. The condition of this patient gradually improved. After being admitted to the hospital for 79 days, he was discharged from the hospital. At this time, the patient had mild dysarthria. The visual of the eyes was restored (the object can be distinguished at 30 cm in front of the eyes). Binocular hyperopia was still poor. Ocular movement was full. The nystagmus disappeared. He could walk less than 5 meters with support. The left upper limb muscle strength was 5-grade on Muscle Strength Grading Scale. The right upper limb muscle strength was grade 3 on Muscle Strength Grading Scale, and the lower limb muscle strength was grade 4 on Muscle Strength Grading Scale. The Babinski’s sign in left was positive, and in right was suspicious positive. The urinary function returned to normal. After discharge, the condition gradually improved. The symptomatic recurred when the steroids were reduced to 1 tablet/day (about 5 months from the first onset). He showed the weakness of the right limb, recurrence of urinary dysfunction. The right limb muscle strength was grade 3 and the bilateral Babinski sign was positive. MRI found multiple abnormal signals in the intracranial, medulla oblongata, spinal, and the spinal cords (Fig. 3). Some lesions were newly added lesions, and some of the original lesions were alleviated or disappeared. A review of a cerebrospinal fluid examination found a pressure of 240 mm H2O, protein concentration of 0.48 g/L, pleocytosis of 14×10^6^/L (multinuclear 0.01, mononuclear 0.99), normal immunoglobulin G, and negative oligoclonal band. The AQP-4 antibodies and MOG antibodies (RIA method) were negative in both cerebrospinal fluid and serum. After giving Human Immunoglobulin (PH4) and steroids pulse again, the condition gradually stabilized.

A 28-year-old male patient was admitted to our hospital due to fever for 7 days, numbness of limbs, unstable walking, and dysuria for 3 days. The body temperature was up to 38.5 °C. At the time of admission, neurological examination revealed clear consciousness and speech, mild horizontal nystagmus, slight hyperalgesia of distal limbs, unstable bilateral finger-nose test and heel-knee-shin test, suspiciously positive bilateral Babinski signs, and normal residual neurological examination. After admission, the condition progressed. On the 4th day, the patient developed diplopia, dysarthria, dysphagia, dyspnea, chest distress, and aggravated weakness of the limbs. He was given a tracheal intubation due to severe hypoxemia. He was on a ventilator. On the 6th day of admission, the heart rate increased and the blood pressure decreased. On the 10th day of admission, the patient’s condition reached a peak. He showed somnolence, urinary retention, bilateral blepharoptosis, bilateral slow pupillary light reflection, limited binocular movement, but normal vision. The muscle strength of his bilateral upper limbs was 1 on Muscle Strength Grading Scale. The muscle strength of his bilateral lower limbs was 0 on Muscle Strength Grading Scale. The limb muscle tension was low. Bilateral pathologic signs were unelicited. Hypoalgesia was found below C5. Brain MRI showed lesions in the dorsal pons and medulla oblongata. A little gadolinium enhancement was found in the medulla oblongata. Cervical spinal MRI showed a T2-hyperintense and discontinuous lesion from C2 to C6 with spot-like gadolinium enhancement. Chest spinal MRI showed a lesion from T5 to T9 without enhancement. Cerebrospinal fluid (CSF) examination revealed pressure of 360 mm H2O, light yellow and transparent cerebrospinal fluid, protein concentration of 4.09 g/L, pleocytosis of 54×10^6^/L (mononuclear 0.99, multinuclear 0.01), and normal IgG. Serum AQP-4 and MBP antibodies were negative. MOG antibody was positive in serum by RIA method (1:32), but negative in the CSF. Other laboratory analysis showed thyroglobulin antibody (A-TG) of 3126.00 IU/ml, anti-thyroid peroxidase antibody (A-TPO) of 209.30 IU/ml, rheumatoid factor of 59.40 IU/ML (normal range: 0-15), immunoglobulin G of 42.30 g/L (normal range: 7.0-16.0), immunoglobulin M of 2.79 g/L (normal range: 0.4-2.3), positive ANA series particle type (1:320), anti-SSA-60+, anti-SSA-52/Ro52+++, serum KAP light chain of 7.86 g/L (normal range: 1.7-3.7), serum LAM light chain of 3.62 g/L (normal range: 0.9-2.1), 24 h urine LAM light chain of 325.12 mg/24 h (normal range<7.80), 24 h urine KAP light chain of 488.32 mg/24 h (normal range<14.20), 24 h urine β2 microglobulin of 139.5 mg/24 h (normal range<0.4), 24 h urine a1 microglobulin of 92.34 mg/24 h (normal range<24), 24 h urine IgG of 104.76 mg/24 h (normal range: 0-17.0), but normal serum β2 microspheres, negative ANCA and antiphospholipid antibodies, and normal serum ceruloplasmin. The immunofixation electrophoresis (IFE) in serum and urine was negative. The complement concentration was normal, and the renal function was normal. The labial glands biopsy showed that the lymphocytic infiltration was <1 lesion. Bone marrow biopsy showed no abnormality. After admission, the patient was given 2 g/kg of Human Immunoglobulin (PH4) (5 times) and steroids pulse treatment. Methylprednisolone was given from 1000 mg/day, and halved every three days until 60 mg/day; then, the steroids changed to oral and slowly reduced. After half a month of treatment, the respiratory function of the patient was restored and the ventilator was stopped. We used intravenous cyclophosphamide 1 g/month to prevent the recurrence of the disease. Patients who were followed up within 6 months of onset had no recurrence of symptoms, but still remained unstable walking, dysuria. At that time, he took oral prednisone 30 mg/day. Neurological examination revealed a normal cranial nerve. The muscle strength of his bilateral upper limbs was 5 on Muscle Strength Grading Scale. The muscle strength of lower limbs was 4 on Muscle Strength Grading Scale. The lower limbs had hypoesthesia of pain and motor sensation. The bilateral heel-knee-shin test was not stable. The bilateral Babinski sign was positive.

## Discussion

The clinical phenotype of MOG antibody disease is diverse, and ON is the most common phenotype of MOG antibody disease, accounting for more than 80%, followed by TM and ADEM. Recently, some clinicians have identified rare cases of MOG Ab-associated demyelinating disease. One case is that the patient can show brainstem encephalitis with pontine trigeminal root entry zone abnormality [2]. The second case is the patient with aseptic meningitis and optic neuritis [3]. And the third case is similar to typical multiple sclerosis [4]. The UK survey found that the clinical manifestations of MOG antibody disease in adults are not the same as in children. In children with MOG antibody disease, ADEM type is more common, accounting for about 40%, and relatively few in adults, about 9% [1]. Adult MOG antibody disease clinical manifestations are closer to the NMOSD. Patients with MOG antibody disease showed a higher count of cerebrospinal fluid white blood cell, content of protein, cerebrospinal fluid pressure, and larger nuclear magnetic lesions [5].

Both of the two patients manifested with the ADEM phenotype, and with typical ADEM clinical features, such as precursor infection, fever, headache, limb dyskinesia, and rapid progression of clinical course, extensive lesions, cerebrospinal fluid pathogens of the virus nucleic acid test was negative, and the serum MOG antibody was positive. However, the clinical manifestations of the two patients are not extremely similar. In case 1, a 15 years old boy showed a sharp decline in binocular vision within 4 weeks of onset and only a light sensation in front of the eye at the most severe course, while in case 2, a 28 years old boy with normal vision, but eye movement combined from the onset. In patients with MOG antibody, the incidence of optic nerve involvement can be as high as 80% or more. Harvard scholars have used transgenic technology to prepare mice that specifically express MOG-specific TCR. About one-third of these mice have spontaneous optic neuritis was found, and if the MOG antigen polypeptide fragment continues to be induced by pertussis toxin, EAE is more likely to occur in the optic nerve than the control group, suggesting that MOG antibodies can specifically invade the optic nerve, which is related to the high content of MOG protein components in the optic nerve myelin [6]. There is a trend that optic neuritis patients with MOG antibody have a younger onset age [7]. In case 1, optic neuritis occurred in the first year form onset, and the fundus examination confirmed the presence of optic nerve changes. In case 2, age 28 years old confirmed no damage to the optic nerve. When optic neuritis occurs in minors, it is necessary to improve the detection of MOG antibodies in order to develop a reasonable treatment plan to reduce the residual and recurrence of visual function defects.

The cerebrospinal fluid characteristics of both patients were similar to viral encephalitis, especially in case 1, the white blood cell count was up to 249×10^6^/L, the classification was mainly mononuclear (0.98), multinucleated 0.02, glucose and chloride were normal. The pressure of cerebrospinal fluids was up to 360 mm H2O. After the steroid therapy, the number of white blood cells and cerebrospinal fluid pressure in the cerebrospinal fluid gradually decrease, while the increase of protein is not obvious. About 50% of patients with MOG antibody have an increase in cerebrospinal fluid albumin and cell count. The number of cerebrospinal fluid cells in patients with 5% to 10% will increase to 100-300×10^6^/L, and CSF Protein > 1 g/L in about 10% of patients [1]. The characteristics of cerebrospinal fluid in MOG antibody disease may be related to the pathogenesis: MOG antigen is expressed on the myelin of oligodendrocytes in the central nervous system only, and there is no MOG protein in the periphery. Although the DNA sequence of the MOG antigen was detected in the genome of the thymic medullary epithelial cells, MOG was not expressed in the peripheral tissues, that is, the peripheral T or B lymphocytes were not tolerant to MOG. When a pathogenic microorganism (usually a virus such as rubella virus, cytomegalovirus, and coxsie virus) invades, the blood-brain barrier is destroyed, MOG antigen enters into the periphery, sensitized T lymphocytes, and sensitized T lymphocytes. The ruptured blood-brain barrier occurred and accumulates around the venules, releasing chemokines and inflammatory signals. Chemotaxis B cells and macrophages accumulate in the inflammation site, causing destruction of the myelin sheath. There is an inflammatory reaction in this process, so the amount of white blood cells can increase in the cerebrospinal fluid, which may be accompanied by an increase in intracranial pressure due to edema of the brain tissue.

The lesions of the brain and spinal cord of MOG antibody disease are often multiple and diffuse. In this paper, two patients had different brain and spinal cord lesions. Case 1 with multiple lesions in the brain, involving the ventral and dorsal medulla, bridge arm, thalamus, corpus callosum, subcortical white matter, cortex, gray matter are also affected, mainly in white matter, away from the lateral ventricle, non-MS-like changes. The lesion volume is large, the average volume is greater than 1 cm, and the enhancement is not obvious. In the case of the patient, the intracranial lesions were limited, involving only the dorsal aspect of the pons and the medulla oblongata. The lesions were located at the bottom of the fourth ventricle. The distribution range was similar to that of the NMOSD intracranial lesions. No lesions were found on the sputum. There are similarities in the lesions of the spinal cord in 2 patients. Although, the affected segments were longer and not continuous. The lesions were punctate or linear, and the lumbar spinal cord was involved. The imaging features of this spinal cord are different from those of MS or NMOSD. The spinal cord lesions of MS are short segments, usually no more than 3 vertebral levels. The spinal cord lesions of NMOSD are usually long segments (more than 3 vertebral levels), continuous lesions, lesions with local edema, and enhancements are small shape. Case 1 with lumbar spinal cord was significantly affected, while MS or NMOSD patients with spinal cord lesions rarely involved the lumbar segment. Combined with the imaging characteristics of these two patients, the intracranial lesions of juvenile MOG antibody seem to be more similar to ADEM-like lesions. The adult intracranial lesions of MOG antibody disease are closer to NMOSD-like intracranial changes. The lesions of the spinal cord are characterized by long segments, discontinuities, and extensive involvement, which may involve the lumbar spinal cord.

MOG antibody disease can overlap with many autoimmune diseases. Jarius et al. found that 42% of MOG-IgG seropositive patients have coexisting autoantibodies, while only 8% of patients have concomitant autoimmune diseases [8]. Some patients with anti-NMDA receptor encephalitis have been reported to have co-occurring positive MOG antibodies [9, 10]. Case 2 combined with a variety of autoimmune antibodies, including anti-SSA-60+, anti-SSA-52/Ro52+++, negative analysis of lip gland biopsy is related to immunotherapy, lip gland biopsy performed 16 days after the start of immunotherapy. The result may be a false negative. After the patient was treated with immunosuppressive agents, the autoantibody titer in the body gradually decreased. Anti-SSA-60 and anti-SSA-52/Ro52 positive, suggesting that the patient may be Sjogren’s syndrome, but the history of the patient, there is no dry mouth, dry eyes, saliva, or tear secretion reduction. In addition, the patient’s serum and urine KAP light chain and LAM light chain were transiently elevated, suggesting that plasma cells are overactive, immunoglobulin synthesis and catabolism are strong, this phenomenon can also be seen in patients with Sjogren’s syndrome. In summary, we analyzed that patients may have multiple autoimmune dysfunctions at the same time, but have not yet reached the diagnostic criteria. After immunomodulatory treatment, some antibody levels decreased significantly or even became negative. The patient still needs a longer follow-up to get a more definitive diagnosis.

Both of the two patients were treated with gamma globulin 2 g/kg plus methylprednisolone 1 g (post-gradual reduction), and the symptoms were alleviated. Case 1 had better motor function recovery, while bladder and rectal dysfunction remained. Most of the symptoms of case 2 were recovered; however, lower limb movement disorder was remained, unable to get up, and indwelling catheterization. Considering the presence of various autoantibodies in the course of case 2, in order to avoid recurrence, cyclophosphamide was added to the treatment, 1000 mg once a month, and the point was static. The patient's condition has been in a period of sustained remission. Case 1 was treated with steroids without the use of immunosuppressive agents. When the prednisone was reduced to 1 capsule per day (after 5 months from the first onset), the symptoms relapsed. The previously reported recurrent MOG antibody disease usually occurs within 6 months from the first onset; however, MOG antibody was not found after the relapse, which seems to indicate that negative of MOG antibody cannot completely used as the exact indication for disease remission, close observation and regular hospital follow-up still needed. Therefore, we do recommend that patients with MOG antibody disease, especially in the 6 months to 1 year after the onset of the disease, steroids should not be reduced too fast, add immunosuppressive therapy is better, and cyclophosphamide is effective.

## Conclusion

MOG antibody disease involves an extensive and complex symptom spectrum, and can be superimposed with various clinical symptoms. It is especially easy to be confused with central nervous system infectious diseases, ADEM, NMOSD, MS, etc. The detection of specific antibodies by the CBA method is diagnostic and prognostic effective methods, and phenotypic differences may also exist in patients of different ethnic groups and regions. The clinical features of MOG antibody-related diseases still require larger samples, multi-center research, and reports.

## References

[1] Jurynczyk M, Messina S, Woodhall MR, Raza N, Everett R, Roca-Fernandez A, Tackley G, Hamid S, Sheard A, Reynolds G, Chandratre S, Hemingway C, Jacob A, Vincent A, Leite MI, Waters P, Palace J (2017). Clinical presentation and prognosis in MOG-antibody disease: a UK study. Brain..

[2] Shen Y, Cheng Z, Zhou C (2019). Bilateral trigeminal root entry zone enhancement in MOG-IgG-associated brainstem encephalitis. Neurol Sci.

[3] Vibha D, Singh RK, Salunkhe M, Dash D, Tripathi M (2020). MOG antibody syndrome presenting as aseptic meningitis: an evolving spectrum. Neurol Sci.

[4] Breza M, Koutsis G, Tzartos JS, Velonakis G, Evangelopoulos ME, Tzanetakos D, Karagiorgou K, Angelopoulou G, Kasselimis D, Potagas C, Anagnostouli M, Stefanis L, Kilidireas C (2019). MOG antibody-associated demyelinating disease mimicking typical multiple sclerosis: a case for expanding anti-MOG testing?. Mult Scler Relat Disord.

[5] Baumann M, Sahin K, Lechner C, Hennes EM, Schanda K, Mader S, Karenfort M, Selch C, Hausler M, Eisenkolbl A, Salandin M, Gruber-Sedlmayr U, Blaschek A, Kraus V, Leiz S, Finsterwalder J, Gotwald T, Kuchukhidze G, Berger T, Reindl M, Rostasy K (2015). Clinical and neuroradiological differences of paediatric acute disseminating encephalomyelitis with and without antibodies to the myelin oligodendrocyte glycoprotein. J Neurol Neurosurg Psychiatry..

[6] Delarasse C, Daubas P, Mars LT, Vizler C, Litzenburger T, Iglesias A, Bauer J, Della Gaspera B, Schubart A, Decker L, Dimitri D, Roussel G, Dierich A, Amor S, Dautigny A, Liblau R, Pham-Dinh D (2003). Myelin/oligodendrocyte glycoprotein-deficient (MOG-deficient) mice reveal lack of immune tolerance to MOG in wild-type mice. J Clin Invest..

[7] Nakajima H, Motomura M, Tanaka K, Fujikawa A, Nakata R, Maeda Y, Shima T, Mukaino A, Yoshimura S, Miyazaki T, Shiraishi H, Kawakami A, Tsujino A (2015). Antibodies to myelin oligodendrocyte glycoprotein in idiopathic optic neuritis. BMJ Open..

[8] Dos Passos GR, Oliveira LM, da Costa BK, Apostolos-Pereira SL, Callegaro D, Fujihara K (2018). MOG-IgG-associated optic neuritis, encephalitis, and myelitis: lessons learned from neuromyelitis optica spectrum disorder. Front Neurol..

[9] Ma J, Jiang L Viral encephalitis followed by anti-NMDAR encephalitis with concomitant MOG antibody-positive central nervous system demyelination in a child. Neurol Sci 41:2303–3033. 10.1007/s10072-020-04357-x 10.1007/s10072-020-04357-x 32200497

[10] Amano E, Machida A, Kanazawa N, Iizuka T (2020). Cerebrospinal fluid MOG-antibodies in anti-NMDA receptor encephalitis with leptomeningeal enhancement. Neurol Sci.

